# Symbiotic Root-Endophytic Soil Microbes Improve Crop Productivity and Provide Environmental Benefits

**DOI:** 10.1155/2019/9106395

**Published:** 2019-04-02

**Authors:** Gary E. Harman, Norman Uphoff

**Affiliations:** Cornell University, Ithaca, NY, USA

## Abstract

Plants should not be regarded as entities unto themselves, but as the visible part of plant-microbe complexes which are best understood as “holobiomes.” Some microorganisms when given the opportunity to inhabit plant roots become root symbionts. Such root colonization by symbiotic microbes can raise crop yields by promoting the growth of both shoots and roots, by enhancing uptake, fixation, and/or more efficient use of nutrients, by improving plants' resistance to pests, diseases, and abiotic stresses that include drought, salt, and other environmental conditions, and by enhancing plants' capacity for photosynthesis. We refer plant-microbe associations with these capabilities that have been purposefully established as enhanced plant holobiomes (EPHs). Here, we consider four groups of phylogenetically distinct and distant symbiotic endophytes: (1) Rhizobiaceae bacteria; (2) plant-obligate arbuscular mycorrhizal fungi (AMF); (3) selected endophytic strains of fungi in the genus *Trichoderma*; and (4) fungi in the Sebicales order, specifically *Piriformospora indica*. Although these exhibit quite different “lifestyles” when inhabiting plants, all induce beneficial systemic changes in plants' gene expression that are surprisingly similar. For example, all induce gene expression that produces proteins which detoxify reactive oxygen species (ROS). ROS are increased by environmental stresses on plants or by overexcitation of photosynthetic pigments. Gene overexpression results in a cellular environment where ROS levels are controlled and made more compatible with plants' metabolic processes. EPHs also frequently exhibit increased rates of photosynthesis that contribute to greater plant growth and other capabilities. Soil organic matter (SOM) is augmented when plant root growth is increased and roots remain in the soil. The combination of enhanced photosynthesis, increasing sequestration of CO_2_ from the air, and elevation of SOM removes C from the atmosphere and stores it in the soil. Reductions in global greenhouse gas levels can be accelerated by incentives for carbon farming and carbon cap-and-trade programs that reward such climate-friendly agriculture. The development and spread of EPHs as part of such initiatives has potential both to enhance farm productivity and incomes and to decelerate global warming.

## 1. Introduction

For more than 300 years, it has been known that there are endophytic microbes which colonize and reside in plant roots. But, only in recent decades, has the value of these microorganisms, both for increasing crop yields and for environmental buffering, become appreciated. Most of the research on these organisms and their effects has been relatively recent.

Symbiotic relationships between plants and microorganisms were first reported in 1697, when Malpighi described the formation of galls on roots. At that time, however, this was more a matter of curiosity than of scientific import. Two hundred years later, Hellriegel and Wilfath demonstrated that these galls are nodules composed of both bacterial (Rhizobiaceae) and plant cells and that they fix N_2_ from the atmosphere, providing leguminous plants with ammonia (NH_3_) as an essential nutrient [1]. In 1882, plant roots were found to be colonized also by fungi that symbiotically enhance the plants' productivity [2]. These organisms are now referred to as arbuscular mycorrhizal fungi (AMF) [3].

In the 1920s and 30s, the very common, soil-inhabiting fungi *Trichoderma* were found to have activity that controls pathogenic fungi, thus having potential to protect agricultural crops [4, 5]. Since then, particular strains of these fungi, but not all, have been found to induce multiple benefits to plants when they colonize roots, more than just protecting against plant diseases [6, 7]. In the 1990s, another group of fungi was discovered that beneficially colonizes and inhabits plant roots, *Piriformaspora indica* [8].

The first three groups of these organisms are now widely used in agriculture, although as discussed here, these organisms have capabilities that are either underutilized or not yet put to use. The fourth group has many uses that are similar to the first three, but it is not as widely employed. These four groups of microorganisms easily and routinely colonize plant roots, and once inhabiting the roots, they induce physiological changes and modify gene expression in the plants that they reside in, thereby enhancing plants' productivity and resilience.

By penetrating and colonizing plant roots, these organisms become part of a symbiotic plant-microbe system. These plants thus should not be regarded as entities unto themselves, existing independently. Rather, they should be regarded and treated as composite organisms.

Plants that exist in association with their microbial colonizers constitute holobionts, assemblages of different species that together form functioning ecological units. Margulis [9] originally proposed this term to describe different microorganisms that combine asexually to create new, integrated hereditary symbionts, but this concept has been expanded to describe more generally host organisms plus all of their microbial symbionts [10]. We are becoming aware that most plants and animals host such microbial symbionts as medical research illuminates the benefits that derive from our human microbiome [11].

The performance and growth of some plant holobionts can be improved if the microbial communities around, on, and in their roots are purposely altered by introducing selected microbial strains or by using other methods that modify the roots' microflora. When plants' microbial component is thus augmented by purposeful intervention, we characterize the resulting composites as enhanced plant holobionts (EPHs), a concept that will be elaborated below.

EPHs can be induced and made more numerous within agricultural production systems by the addition of carefully selected members of any of the four groups of microbes reviewed in this paper. While there are other microorganisms that can play similar roles as plant symbionts, we focus on these four groups of organisms which are well studied and well documented in the literature.

This review paper examines how the benefits of these microorganisms can be capitalized on both for our human species and for the sustainability of our natural environment. The advantages that EPHs have over conventional plants, even when the latter have been endowed with state-of-the-art genetics, include the following benefits:

1. *Enhanced plant growth*, including larger and deeper root systems
2. Greater and more-assured yields
3. *Enhanced uptake and more efficient use of nutrients*: in the case of Rhizobiaceae and the legumes that they nodulate, there is a direct increase in the plants' supply of nitrogen through the acquisition of N_2_ from the atmosphere and its conversion into plant-available NH_3_
4. *Enhanced resistance to numerous plant pests* that reduce crop yield, including pathogens, nematodes, and herbivorous insects
5. *Enhancement of plants' resistance to abiotic stresses*, including drought, salts, and soil pollutants
6. Enhanced rates and levels of photosynthesis

These capabilities make EPHs desirable for a world that by 2050 will require the production of at least 50% more food, while agriculture is constrained by diminishing per-capita resources of land and water and by foreseeable climate change.

This review and synthesis of knowledge will

1. Present *four groups of endophytic microbes*, describing their respective *lifestyles* as plant symbionts and the *mechanisms of action* that they have in common
2. Discuss and describe the abilities of EPHs to *suppress or control plant pests*, including plant-pathogenic microbes and viruses, nematodes, and herbivorous insects
3. Review the abilities of EPHs to *alleviate stresses on plants* caused by drought, salts, or chemical pollution of soils
4. Describe the processes of strain selection, production, and formulation of *beneficial microbial applications* and the agronomic practices that can enhance their activity
5. Consider the uses of EPHs to *sequester CO*_*2*_*from air while increasing agricultural productivity and alleviating stresses on crops*, transferring C into soil, and thereby countervailing present increases in atmospheric levels of greenhouse gases

## 2. Four Groups of Endophytic Microbes, Their Lifestyles as Plant Symbionts, and Mechanisms of Action that They Have in Common

The four groups of symbiotic microbes considered here are (a) bacteria in the family Rhizobiaceae; (b) arbuscular mycorrhizal fungi (AMF) in the phylum Glomeromycota; (c) specific strains of fungi in the Ascomycetous genus *Trichoderma*; and (d) fungi in the order Sebicales, exemplified by *Piriformaspora indica*. Although these organisms are phylogenetically distant and distinct, each group has independently evolved means to internally colonize plant roots, thus becoming resident plant root endophytes.

These four groups are true plant symbionts in that they confer advantages to the plants whose roots they colonize, while at the same time, they derive nutrients and other benefits from their plant hosts [7]. Although their infection mechanisms and lifestyles within plant roots differ, the advantages that they confer to plants are similar. There are large numbers of other microbes that are associated with roots [12], but these four groupings have documented capabilities to serve as components of EPHs.

### 2.1. Rhizobiaceae

Bacteria in the Rhizobiaceae family show complex interactions with the leguminous plants whose roots they nodulate. Infection occurs through plant root hairs in conjunction with complex plant-microbe chemical signaling. In many cases, the plant produces “infection threads” that guide the bacteria to plant root cells where the bacteria morph into nitrogen-fixing bacteroids [13]. In other cases, the bacteria simply infect the roots through surface cracks formed where secondary roots have emerged [14].

Leguminous plants produce complex nodules around the bacteroids' nitrogen-fixing cells. These nodules are filled with the iron-containing protein leghemoglobin that removes oxygen, thus providing the hypoxic environment necessary for the bacteroids to fix nitrogen, reducing N_2_ to NH_3_, a form of nitrogen that can be readily utilized by the host plant [13]. The interactions that lead to this nodule formation are highly specific, with only certain rhizobial species or strains able to colonize a particular host legume [15].

It is less well known that Rhizobiaceae are also able to colonize the roots of many nonleguminous plants, including cereals [16–20] and potatoes [21]. This infection occurs directly between epidermal cells or through root cracks. In the case of cereals, after establishing themselves in the plant roots, these bacteria ascend into the upper portions of the plant where they confer significant advantages for plant growth and development [22].

Rhizobiaceae thus have both a highly specific plant-microbial interaction that leads to nitrogen-fixing nodules in legumes and a much less host-specific mechanism that benefits many nonleguminous plants. Both mechanisms are highly advantageous to the host plants.

### 2.2. Arbuscular Mycorrhizal Fungi (AMF)

These fungi are obligate plant symbionts, which means that they cannot be cultured and cannot grow unless a plant host is present. These fungi are the only organisms considered in this review that are obligate symbionts, dependent on their plant hosts for survival. AMF form associations with most terrestrial plant species, and the infection and establishment of these fungi within plant roots is a complex process, involving intricate chemical signaling between the fungus and its host. Both mycorrhizal and rhizobial associations require complementary microbial and plant genes that together establish a symbiotic genetic pathway which was first evolved within AMF about 400 million years ago [23].

Once the fungi penetrate the host roots, a prepenetration apparatus (PPA)—analogous to the infection threads used by Rhizobiaceae—guides the fungi to an appropriate cell determined by the host [24, 25]. Once located within plant root cells, AMF form relatively large lobed structures (arbuscules) that are located between the host's cell wall and the plant cell membrane [25]. The size of the plant's cell membrane is thus increased several-fold by conforming to the surface of the lobed arbuscule [24, 25]. Enlarging the surface area of the contact region provides a much greater area for the exchange of materials between the fungus and its host plant's cells.

### 2.3. Selected *Trichoderma* Strains

Fungi in the genus *Trichoderma* are among the most widespread in the world, being probably the most commonly isolatable fungus in soils [26–28]. Members of this genus have many diverse lifestyles, including saprophytic growth in soils in which they degrade many complex substrates, particularly those with cellulose, chitin, and lignin components. *Trichoderma* are not limited to soil environments and may colonize also trees and branches, including epiphytic colonization of tree stems and shoots [29, 30]. Some strains are able to colonize plant roots via direct penetration of the plant cell walls, and a few become highly efficient endophytes that persist for the life of the plant [6, 7].

### 2.4. *Piriformaspora indica*

These fungi directly penetrate cells and establish colonies within them. Their growth within roots is accompanied by programmed plant cell death, and the fungus proliferates within these killed cells [8, 31]. The established fungus is located within the root's cortical cells but does not colonize or damage meristematic cells [31].

#### 2.4.1. Commonalities

With all four kinds of infection, root colonization establishes a mutually beneficial interaction with the host plant. Some of these beneficial interactions such as nitrogen fixation of rhizobia with legumes or enhanced phosphorus uptake as a consequence of infection with AMF [25] are well known and widely utilized. Other beneficial interactions are less recognized and little exploited in agriculture.

In most of the interactions described above, the microbial partner is restricted to residence in the plant root. Yet, in every case, its effects on the plant are systemic, with numerous changes occurring in the plant's gene expression and protein expression and consequently in its phenotype. Remarkably, the changes induced in plants' gene expression may be more numerous and have greater effect in the above-ground portions of the plant than in the roots [32]. Affecting system-wide changes in plants' gene expression requires that these microbes in the root produce chemical signals that are transmitted to the rest of the plant, thereby altering crop phenotypes.

#### 2.4.2. Chemical Mechanisms

Each of these four groups of organisms produces molecules that function as signaling molecules within plants. We refer to these molecules collectively as *symbiont-associated molecular patterns* (SAMPs), a subset of the signaling molecules previously designated as microbial-associated molecular patterns (MAMPs) [33]. While these molecules differ between the four groups, all apparently interact with receptors in the plant cell's plasmalemma. Such interactions are essential to permit infection and plant receptivity in the specific Rhizobiaceae-legume interactions leading to nodulation and to root infections with AMF [13, 24, 25].

All four groups of microbial symbionts produce SAMPs that are required for systemic effects within plants which are temporally and spatially distant from the site of application of the symbionts. These molecules include diverse lipochitooligosaccharides that are produced by Rhizobiaceae and AMF [34–36] (and structurally related to chitooligosaccharides [34]); cellotriose produced by *P. indica* [37]; and a diverse group of SAMPs produced by *Trichoderma* strains that include volatile and nonvolatile small molecules [38, 39] as well as peptides and proteins [40–42] (Table 1).

The physiological changes and enhanced plant capabilities which are induced will be addressed in the following sections. In many cases, systemic and long-lasting changes can be evoked either (a) by treating the plants just with the SAMP or (b) by inoculating the plants with the organism that produces these molecules. SAMP recognition by the plant provides inducible signals, probably transduced by MAP kinases, with the consequence that plants' gene expression is reprogrammed [51, 66].

This reprogramming, at least in part, involves changes in gene regulation. This can occur by alterations in the cell's chromatin; by modification of the histones or by DNA methylation in the upstream regulatory portions of genes [67]. These changes can result in what is referred to as “gene priming,” a process whereby gene products are not expressed until these products are needed, such as in response to abiotic or biotic stresses. In a “primed” state, genes are expressed more rapidly and more fully in response to whatever is the stimulating factor [68–70].

## 3. Benefits Provided by EPHs

Understanding the evolved processes and relationships that exist in nature can enable humans to initiate changes in plant-microbial symbiosis, by introducing more or different microbes that become part of the plant's microbiome. This is the basis for the proactive enhancement of plant symbionts. Some of the best understood advantages that they confer are reviewed in this section. Additional benefits are discussed in Section 5.

### 3.1. Plant Growth Promotion

The benefits of N fixation in legumes and the enhanced uptake of soil nutrients as a consequence of root colonization by microbial symbionts have long been known. Seed treatment of legumes with Rhizobaceae frequently leads to yield increases [43], especially when newer strains and formulations are used which are more efficacious (see Section 4.3). The greatest yield increases occur when inoculated seeds are planted in soil where rhizobia are not already established; but even in soils that have been colonized by rhizobia, yield increases can occur, albeit not as great [44] (Table 1). Millions of acres are already being planted with leguminous plants such as soybeans inoculated with appropriate rhizobia. The current market value of such products is estimated to be over $230 million [71].

While much of the current market demand stems from the benefits of nitrogen fixation in legumes, these bacteria can also colonize other crops that have no nodule formation. In the *Rhizobium*-cereal model, the same bacteria that fix nitrogen can colonize the roots of nonleguminous plants through crack infection (Table 1) [17–20]. After the microbes proliferate in the roots of rice, wheat, and other cereals, they ascend into the upper portions of these plants. This colonization results in increased growth and yield of many kinds of crop plant [22].

This effect is associated with changes in gene expression. A transcriptomic study of rice seedlings [72] identified 2,414 differentially expressed genes (DEGs) during the plants' first eight days after the seedlings had been inoculated with *Sinorhizobium meliloti* 1021. Changes in gene expression occurred even before the bacteria had colonized the upper regions of the plants, so this showed systemic effects [72]. DEGs of particular importance included the upregulation of specific genes involved in photosynthesis [72, 73]. Other DEGs identified included genes involved in cell wall modification, cell division, plant signaling, transport within plants, carbohydrate metabolism, and nucleotide signaling [73].

A critical need for crop improvement to meet the anticipated food needs in the coming decades is to achieve greater photosynthetic efficiency [74]. As reviewed below, symbiotic microorganisms can enhance this capability in particular through a reprogramming of plants' gene expression [32, 49, 51, 72, 73].

Recently, there have been bioengineering attempts to enhance some plants' photosynthetic efficiency, for example, redesigning tobacco chloroplasts to contain microcompartments (carboxysomes) [75] or engineering synthetic photorespiratory bypass systems into the same plant [76]. The latter approach has been shown to improve plant yields. However, bioengineered plants face both developmental and regulatory issues, and it will be some time before they are ready to use in the field; e.g., each individual variety and crop species has to be engineered individually. Conversely, the use of endophytic symbionts requires no plant genetic modifications or any introduction of heterologous genes, and EPH systems are already available for use.

AMF are ubiquitous colonists of plant roots. Without them, many plants, especially those living under adverse conditions, would not be able to survive [77]. However, we note that a meta-analysis of yield with and without added AMF in intensively managed field crops has indicated little yield benefit from the augmentation of AMF populations (Table 1). This was at first surprising because plant systems have coevolved with diverse endophytic microflora, starting with mycorrhizal interaction that arose around 400 million years ago [23].

Unfortunately, AMF may provide only limited benefits within contemporary farming systems that are based on repeated and major soil disruption [24, 47, 48]. This is partly because these organisms as obligate plant symbionts cannot easily be cultured and produced in pure form on an industrial scale for application to large-scale row-crop farming. It is well documented that they can increase the uptake of nutrients from the soil, especially phosphorus [3, 24, 25], can induce resistance to plant pathogens [78–80], and can reduce abiotic stresses [49, 81–83]. They are beneficial in perennial and other agricultural systems where soil disruption is minimal. AMF provide advantages that are important and may be best enhanced using carbon-farming systems described in Section 5.2, rather than through direct application to intensive row-farming practices.

**Figure 1:**
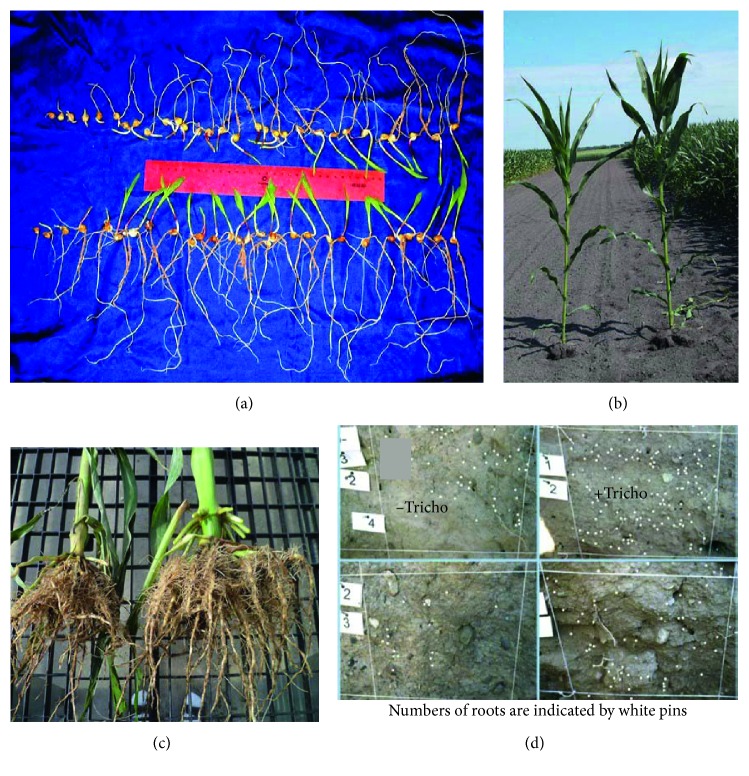
Plant endophytic symbiotic microorganisms are able to enhance plant growth and development from seedlings to maturity, as evidenced by these examples from the use of *Trichoderma* with corn. (a) Ten-day-old seedlings of an inbred maize line (Mo17) grown from untreated seeds (upper row) or from seeds treated with *T. afrohazianum* (lower row). The differences in size that are seen in the seedlings persist in the mature plants. (b) Appearance of corn plants in a commercial trial in Minnesota. The plant on the right was grown from a seed treated with a commercial product containing *T. afroharzianum* and *T. atroviride* overtreated onto a standard chemical pesticide, while the plant on the left grew from a seed treated only with a chemical pesticide. Photo courtesy of Advanced Biological Marketing. (c) Both the organisms and their SAMPs can induce season-long changes that affect both shoots and roots. Shown are roots of mature corn plants grown from either seeds treated only with a chemical pesticide (left) or with similar seeds overtreated with the SAMP 1-octen-3-ol at picoliter quantities (right). The observed increase in root growth is distant both temporally (several months later) and spatially from the site of application of the SAMP. Photo courtesy of Advanced Biological Marketing. (d) *Trichoderma* strains increase rooting depth. Corn plants were grown in the field to maturity, and then, trenches were dug adjacent to them about 2.3 m deep. The soil faces next to the plants were treated with a power washer to expose root intercepts and were marked with map pins that show up as dots in the figure. At 25–75 cm below the soil surface, there were about twice as many roots from plants grown from *Trichoderma*-treated seeds as from untreated seeds [6].

Both *P. indica* and selected strains of endophytic *Trichoderma* colonize the roots of most plants. Once they enter the roots, they can persist for the life of at least an annual crop (Table 1), frequently enhancing the growth of shoots and roots (Figure 1). They also induce crops' resistance to diseases and pests (Table 2) and reduce plants' symptoms of abiotic stress. These effects involve the activation of internal systems which ameliorate the toxic effects of ROS and which increase plants' photosynthetic capabilities (see following sections). This makes them highly versatile plant symbionts.

*Trichoderma* strains, in contrast to AMF, are easily propagated and are widely used in agricultural production systems around the world. Recent reviews of the number of products for agricultural use that are based on these fungi range from 27 to 60 [30, 91]. The products sold include one or more of the >10 different species that have demonstrated enhancement of crop productivity. *Trichoderma* products are widely distributed, with the largest number sold in Asia [91]. However, even though some of these microbes, especially Rhizobiaceae and *Trichoderma*, are now fairly widely used, their uses and capabilities are not yet fully exploited.

### 3.2. Plant Disease and Pest Control

The endophytes considered here all have the ability, primarily through the induction of systemic resistance, to control, i.e., reduce if not eliminate, many plant diseases and pests. Examples are given in Table 2.

Rhizobia are best known for their N fixation in the nodules on legume plant roots, as noted above. However, their biocontrol capabilities are also now well documented. Their mechanisms of action may include mycoparasitism through the colonization of the bacteria within fungal pathogens [85] (Table 2). Rhizobia may also produce antibiotics such as bacteriocins (proteinaceous or peptic toxins) or hydrogen cyanide that inhibit the development of pathogens. Also, rhizobial production of siderophores can limit undesired fungal growth by efficiently sequestering metal ions needed by the pathogens for their growth. Further, the endophytic presence of rhizobia may also increase or activate plants' internal mechanisms for systemic resistance.

A number of pathogens, primarily but not exclusively living in soil, have been demonstrated to be controlled by rhizobia (Table 2) [84]. In at least one case, this resulted from the colonization of roots of a nonleguminous plant, Indian mustard, by a strain of *Mesorhizobium loti* which conferred a high level of control of the white rot organism that adversely affects the roots and lower stems [86]. The high species specificity of rhizobia with regard to legumes may be primarily for its nodule formation and not necessarily for plant colonization more generally, as was described in the discussion of cereal-rhizobia interaction above.

AMF have been shown to control a wide range of plant pathogens, including fungi, oomycetes, bacteria, viruses, and nematodes. For example, when wheat roots were colonized by AMF (*Funneliformis mossease*), the levels of powdery mildew on crop leaves were reduced by almost 80%. Since the AMF are located only in the plants' roots, the protection conferred on leaves had to be a consequence of induced systemic resistance. Indeed, analysis of wheat's gene expression indicates that there was upregulation of certain genes for plant defense and also for the production of antifungal phenolic compounds and H_2_O_2_ [87] (Table 2).

Potato plants whose roots had been colonized by *Rhizophagus irregularis* have been found to lower levels of the potato late blight caused by *Phytophthora*, and the symptoms occur later. However, under higher disease pressure, no reduction in symptoms was observed [78]. This is not an unusual result. With high disease pressure and intense exposure to pathogens, adequate control may require the use of a chemical pesticide and/or optimization of the holobiont's biological system to attain adequate results, e.g., through early application of the symbiont before the pathogen infects the plant or the disease occurs.

Plant resistance to viruses may also be induced by beneficial root endophytes. Symptoms of tomato leaf curl have been found to be milder, and the levels of virus DNA reduced, by the presence of *F. mosseae* [88]. Another report has extended the range of pathogens that can be controlled by symbionts to include bacteria-like organisms that lack cell walls, the aster yellows phytoplasma, and *Spiroplasma citri*. These pathogens when living in the plant's phloem can cause significant disease for a medicinal plant, the Madagascar periwinkle. Colonization of its roots by *Glomus mosseae* gives the plant resistance against *S. citri*, although this fungal microorganism was not found to control the aster yellows disease [79]. A recent summary [80] lists a number of tests in which AMF were effective in nematode control and/or for increasing yields when compared to plants that were infested with these roundworms but had no biocontrol applications (Table 2).

Root colonization by *P. indica* protects plants against various diseases. These include the root rot pathogens *Verticillium*, *Fusarium* foot rot, *Rhizoctonia* and *Thielavopsis* [31], and several fungal diseases of roots and stems, including *Alternaria brassicae, Botrytis cineria* [89], and powdery mildew [51]. In addition, *Fusarium* head blight has been reduced in wheat, and the levels of the mycotoxin deoxynivalenol minimized, by *P. indica* colonization of wheat roots when head blight and its associated mycotoxins were a serious problem [90].

*P. indica* acts by inducing systemic resistance. Evidence of this is seen in its ability to control a variety of pathogens that infect above-ground stems, leaves, and the developing grain [31, 51, 90]. This endophyte does not appear to produce antibiotic substances [89], rather it induces plants to produce certain metabolites and enzymes that ameliorate the effects of reactive oxygen species (ROS) [51, 89, 92]. ROS molecules are produced within plants in response to a variety of biotic and abiotic stresses. As seen below, these unstable molecules are highly damaging, causing browning of leaves and adversely affecting plants' photosynthetic organelles.

*Trichoderma* species were first considered for use in agricultural production as biocontrol agents against plant diseases, based on their abilities to parasitize other fungi and produce antibiotics. However, their primary mode of action for plant protection is now known to be by the induction of disease resistance [7]. *Trichoderma* have documented abilities to control many deleterious organisms, including other fungi (e.g., *Fusarium, Curvularia*, *Colletotrichum, Alternaria, Rhizoctonia*, and *Magnaporthe*), oomycetes (*Pythium* and *Phytophthora*), bacteria *(Pseudomonas* and *Xanthomas*), and at least one virulent virus (green mottle mosaic virus on cucumber). Summaries of these several capabilities have been published [7, 93], and examples are given in Table 2.

Evidence for the induction of systemic resistance [7, 93] is seen from the fact that many diseases caused by the pathogens just noted are diminished in the above-ground plant parts even when the symbiont has only infected the plant's roots. Moreover, mutants of *T. virens* which are not themselves mycoparasitic and/or lack antibiotic activity, nevertheless, have the ability to control *Rhizoctonia.* This is closely correlated with these strains' ability to induce plants' production of terpenoid phytoalexins, which are strongly antifungal [7, 94]. Disease control is induced in the plant by getting it to produce antifungal compounds, a concrete example of induced resistance.

The ability of *T. harzianum* to control *Pythium* requires that the plants possess a functional *NPR1* gene. This is an early regulatory gene required for evoking plant resistance by either the jasmonate/ethylene or salicylate-induced resistance pathways [58].

Biological control of the root-feeding nematode *Meloidogyne hapla* is induced at least in part through systemic resistance. This is seen from experiments that split the root systems of tomatoes into two with the plant still having a single stem above them. One-half of the root system was in soil that contained both *Trichoderma* strains and nematodes, while the other half was in soil containing only nematodes. Almost 50,000 nematode eggs were laid on the roots of control plants that had had no *Trichoderma* treatment. On the split-root plants, when using the most effective strain of *Trichoderma* for the treatment, the number of nematode eggs laid on or near the roots was about 1,000—only 2% as many eggs were laid on the roots of treated plants as on the control roots. There was no significant difference in laying of eggs on the roots between the side with *Trichoderma* + nematodes and the nematode-only side (with no *Trichoderma*). These data suggest that some chemical signal was being translocated through the single stem to the nematode-only side that inhibited the life cycle of these roundworms. The result was strongly influenced by the choice of *Trichoderma* strain or the mixture of strains [56].

All of these data support the proposition that induced resistance is the primary mechanism for these productive fungi to achieve endogenous biological control of diseases. Moreover, it is known that SAMPs from selected microbial strains can by themselves induce resistance in field plantings. For example, in grapes, either foliar application or a soil drench with the biochemicals harzianic acid or 6 pentyl-*α* -pyrone is able to induce resistance to foliar powdery mildew [61] (Table 2). LCOs and COs applied to plants at low concentrations induce both plant resistance [36] and increased yields [34].

#### 3.2.1. Systemic Resistance

Induced resistance typically is mediated by plant hormonal signaling. Either of the two principal pathways may be initiated. The first, known as *systemic acquired resistance* (SAR), uses salicylic acid as a principal signaling molecule. The second, *induced systemic resistance* (ISR), involves jasmonic acid and ethylene as signaling molecules. There is a considerable amount of cross-talk between these two pathways, and there can be variants of each. The interactions and respective triggering systems are provided diagrammatically in [7].

In summary, the symbiotic organisms that are described here as well as some others [95] have been shown to have substantial efficacy for the control of plant diseases in laboratory or greenhouse evaluations as well as in the field. These biological systems are fundamentally different from the synthetic chemical-dependent approaches to plant disease control currently promoted.

Almost all chemical pesticides operate with a lethal mode of action that directly kills the pathogen. In contrast, biological controls are more subtle, regardless of their mode of action. They act first of all to increase the health of the plant through enhanced nutrition (nutrient acquisition from the soil and/or nitrogen fixation). Then, they can counter pathogens through parasitism or antibiosis. A third line of defense is through the induction of systemic resistance in the plant. None of these biological mechanisms is absolute. They serve to limit the disease and its spread.

Because of the lethal action of pesticides, these chemicals exert strong pressure on the pathogen or pest population to mutate or to change to make it less susceptible to chemical extermination. Biological controls, on the other hand, do not eliminate or kill the pests. Resistance to biological means of control is thus much less likely to develop than in response to chemical pesticides.

For example, a study of resistance to foliar diseases that was induced by *Trichoderma* strains showed 40–80% reductions in the severity or incidence of disease but not 100% protection [7]. This induced resistance is frequently long-lasting, persisting for an entire season, but it is not absolute. A basic strategy when using the root-colonizing symbionts reviewed here is to grow plants that have early and sustained improvements in health and vitality. The processes conducive to this have been discussed above and are discussed further in following sections.

It may seem to be a truism that healthier, more robust plants will suffer less disease and will be more resistant to abiotic stresses. The advice of “growing healthy plants” to control or curb disease is not tautological, however, even if it might appear to be. Biological protection is preventive or ameliorative rather than curative. Chemical pesticides are frequently applied to plants after a disease has been observed. For biological measures, it is important that these be applied early in the life cycle of the plant, establishing a healthy and robust plant that has better intrinsic defenses against pests or pathogens. Making the plant less likely to become ill minimizes the need for intervention with chemical pesticides [6].

Moreover, induced systemic protection has been shown to be beneficial for dealing with diseases for which chemical pesticides are not effective or not efficient. For example, farmers' ability to control nematodes with chemical pesticides is becoming limited because many soil fumigants have been banned, due to their toxicity to nontarget organisms. Also, chemical means for controlling nematodes and root rot are frequently infeasible because such a large mass of soil must be treated for them to be effective. For diseases that have been difficult to control with chemical pesticides such as wheat head blight, resistance-inducing symbionts can be particularly useful means, but there has been little use of such applications thus far.

It is sensible to look to fungal and bacterial communities for means to deal with many if not all crop-protection challenges. This makes sense especially for certain viral and bacterial diseases that are inherently difficult to control through chemical means. Since beneficial microbial communities are self-assembling and for the most part self-sustaining, very small amounts applied as seed treatments or by other means can be highly economical. Opportunities and benefits from using such means are currently underexploited.

## 4. Requirements for Successful Use of Endophytic Microbes

### 4.1. Strain Selection and Identification

Successful use of these beneficial organisms requires that the strains of each be tested and selected for their effectiveness under specific conditions before any widespread use. There can be tremendous variation between different strains of the same species of microbial endophytes, and efficacy can be conditional on various factors. *Trichoderma* strains may be an extreme example of this variation. The first author has direct experience of this. Of the tens of thousands of *Trichoderma* strains that he evaluated over three decades, only six when tested were found to be sufficiently effective in the field to be deployed extensively for agricultural use. Three of these were variants of *T. afroharzianum*, one was a *T. viride*, and two strains were *T. atroviride* [6, 56, 93, 96, 97].

The importance of finding and selecting effective strains is demonstrated by the fact that *Trichoderma* spp. are widespread throughout crop ecosystems all around the world [29]. The total numbers of this fungus already residing in any field soil will greatly outnumber those being added by treating seeds with symbiotic strains. The numerous wild strains evidently do not enhance crop yields or have a beneficial endophytic lifestyle with crop plants.

If they did, the strains that can be added through seed treatment would probably not provide the advantages that we can demonstrate experimentally since the native microflora would be at a competitive advantage. They could therefore control any symbiosis-driven improvements in plant performance. Examples below demonstrate the strain-specificity of highly efficient root colonization that induces systemic changes in crop plants' physiology.

Because these effects have little specificity to plant species [30], the choice of strains for inoculation has to be based on extensive testing. Whether the strains of *Trichoderma* are modified or native, only a select few provide the benefits that are reported throughout this paper.

Strain-specificity occurs not only with *Trichoderma* strains. For example, a strain of *Rhizobium* that is capable of enhancing rice plant growth was found to increase the growth of maize in only 1 of the 10 crop genotypes tested [19]. Similarly, some genotypes of maize do not respond to a particular strain of *T. afroharzianum* or may even respond negatively [98].

Even when soils have been previously cropped with particular legumes that contain beneficial rhizobacteria, extension agents generally recommend that farmers reinoculate their crop each year since this will do more to increase yields [44, 45]. Reinoculation enhances depleted levels of the nitrogen-fixing organisms in the soil, recognizing that Rhizobiaceae are quite sensitive to their environment (see Section 4.2). Also, frequently, the strains that are available commercially have been enhanced by some genetic manipulation so that they can give superior results (see these patents, for example, [99–101]).

To be optimally productive and profitable, strains may need to be locally adapted, although some strains are already being effectively used on multiple crops worldwide [102]. Which strains will be most suited to local conditions, however, is best understood by local testing [43].

One limitation on the use of mycorrhizal fungi is that the selection and genetic manipulation of AMF strains is difficult because they are obligate plant symbionts. The authors are unaware of any efforts to select or manipulate strains of *P. indica* thus far, although this could prove beneficial.

### 4.2. Considerations for Production, Formulation, and Use

The widespread use of endophytic organisms will be broadly affected by the processes that are used for producing the microbial agent and by the resulting quality of the product. The costs of production inputs are always a consideration for farmers because prices affect profitability, especially in the production of high-volume crops like corn. Acceptable levels of cost for small-scale vs. international-scale EPH systems are likely to differ markedly [102]. Also, good quality control is always needed in the manufacturing process so as to produce the desired organisms with requisite concentration and purity. Some of the factors that go into quality manufacturing of *Trichoderma* strains are discussed in [103].

A major limiting factor in producing any organism for application is the difficulty of excluding contaminating microbes from the product. Also, the material can have inconsistent quality in terms of its concentration and its appropriateness for the physiology of the organism being multiplied. Varying quality of formulations of inoculating materials will contribute to erratic results in the field, which can discredit and deter the further use of endophytic treatments.

The nature of the microbes being used and the respective factors that affect the large-scale production of each must be taken into account and allowed for if the organism is to be successfully used in commercial agriculture. Rhizobia, being Gram-negative bacteria like many other eubacteriales, exist only as vegetative cells and do not produce spores or other resting structures. As a consequence, the shelf-life of prepared commercial products that utilize these organisms may be very short.

#### 4.2.1. Applications of Endophytes

The most common method for utilizing microbes is seed treatment. For such use, products have often been prepared as a powder that contains the biological materials. For example, powdered peat has often been used as a medium in seed applications. In the past, however, these formulations have had a very short shelf-life, especially for rhizobia and other Gram-negative bacteria, so that the seeds needed to be treated just before planting.

There have been advances in the development of endophytic formulations, such as encapsulating the cells in a protective coating, which extends product shelf-life [104]. The development of stable liquid formulations is preferred for use in large-scale seed-treatment equipment and has been accomplished with some commercial products (http://www.abm1st.com). Such advances have made possible the development of more effective seed treatments that can be applied in commercial treaters, giving the microbes at least one year of life on the seed. This technical innovation has supported the commercial use of symbiotic organisms over millions of hectares in commercial agriculture.

Care must be exercised in the choice of soils where these treatments are used since many factors will affect the treatments' efficacy and results: soil organic matter (SOM), nutrients, pH, salinity, and agricultural practices, e.g., organic management, no-till cultivation, rotations, and application of pesticides. Temperature and drought-conditions will also have an effect [104].

#### 4.2.2. Special Considerations for Arbuscular Mycorrhizae

The use of AMF is limited by the fact that they are obligate plant symbionts or commensals, as discussed above. This restricts the development of new strains and the modification of existing strains. It also limits their commercial use. Since most AMF products are mixtures of plant root fragments that contain these fungi, this makes it very difficult to prepare products that are not contaminated with some other organisms. It also is difficult to reliably prepare AMF products with high levels of the active ingredient. This has limited the large-scale use of products containing AMF, and at present, most are sold to home gardeners or to organic growers, with limited use in commercial agriculture [24].

AMF can be highly effective in enhancing plant productivity and especially in improving plants' nutrient uptake where extensive fungus-plant hyphal networks can be established. This means that their best use is usually in undisturbed perennial-plant applications such as forests and pastures. Several considerations limit the efficacy of AMF products as inoculants or additives in row-crop agriculture: the prior use of soil fumigation chemicals, the low or nonresponsiveness of certain crop plant varieties to AMF, crop rotations that are based primarily on nonmycorrhizal crops, or some crops' low response to AMF [104]. The obligate nature of AMF is a constraint on the development of improved strains and on efficient large-scale production. Genetic studies and the manipulation of these fungi are thus constrained by their innate plant-dependence [8].

#### 4.2.3. Other Considerations for Endophytic Applications

*P. indica* has been developed for agricultural use in part to circumvent the difficulties of producing highly effective AMF applications given the obligate nature of this fungus. Because *P. indica* has evident capabilities for enhancing plant productivity and performance, it should be possible to produce this endophyte in large quantities [8]. However, the commercial use of this organism has been impeded by various obstacles in production, including the toxicity to plants of some *P. indica* preparations [105]. Even so, the potential of this organism for enhancing crop productivity is clear from the benefits that have been reviewed here.

As noted earlier, certain strains of *Trichoderma* have been proposed for use in commercial agriculture for more than 80 years, primarily for the biological control of diseases and pests [4]. Appreciation of the broader ability of some strains of this fungus to colonize roots and act as endophytic symbionts is more recent [7, 106].

*Trichoderma* reproduce by producing spores (either conidia or clamydospores) that several companies have been able to produce in large quantities. With proper systems of multiplication, levels of 10^9^ to 10^10^ active propagules/g can be produced in a short period of time. These can be used either as seed treatments or as soil treatments on a wide range of crops [6]. Unlike the other organisms considered in this paper, they can be used also as foliar applications for disease control and to provide growth benefits to plants [61, 107]. The characteristics of the cells of the other three groups reviewed here largely preclude their foliar use, although *P. indica* could possibly be used in this mode of application if properly formulated.

It can be confusing that different strains of *Trichoderma* will provide different benefits to crop plants, as will the physiology and the purity of the materials produced. Endophyte products can be manufactured and sold as seed treatments, as greenhouse soil amendments, as soil or transplant drenches, or as foliar treatments. The products may be intended for just local distribution and use, or they may be produced to serve international markets. The criteria and rationale for such products can differ markedly between local and large-scale production [102]. Marketing patterns and structures for *Trichoderma* are still evolving.

**Figure 2:**
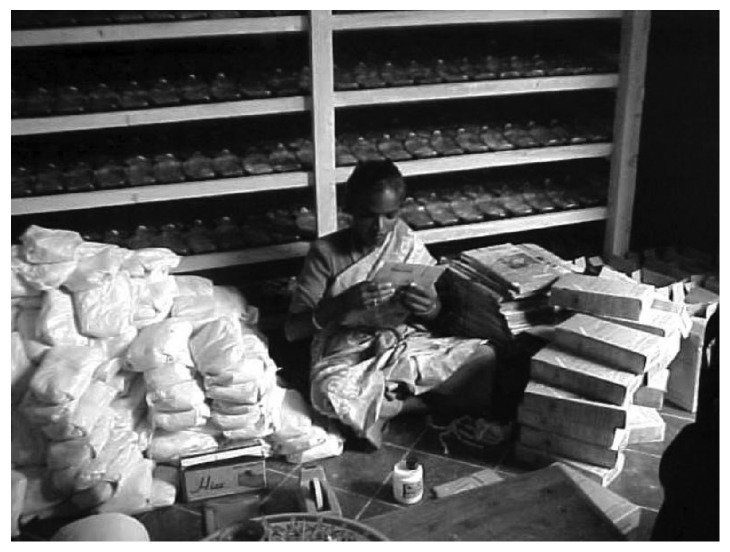
Packing of *Trichoderma viride* biofertilizers in a village production center in Tamil Nadu state of India initiated by the M.S. Swaminathan Research Foundation in Chennai [109].

Criteria, formulation methods, and distribution are bound to vary considerably for the different classes of endophytes, depending on the type of market as well as the kind of agriculture for which they are being produced [102]. The M. S. Swaminathan Research Foundation in India has been able to establish village-level production of *T. viride* as a biofertilizer manufactured by village women in Tamil Nadu State [108] (Figure 2). Other cottage-industry models are being developed. *Trichoderma* can be produced locally for immediate use as much as a one-day's car-drive away. In some cases, several microbial agents have been produced in liquid fermentation systems and applied through a fertigation system directly to the crop [102]. These systems have been successful but are not yet suited to large-scale production. No regulatory approvals were required. On the other hand, for international production, distribution, and registrations in multiple countries, investments of several million dollars will be necessary to cover regulatory, development, and large-scale manufacturing costs [102].

One advantage of *Trichoderma* is that many of its strains are resistant to many of the commonly used pesticides and thus can be applied as an overtreatment on chemically treated seeds. Also, they can be used in integrated biological-chemical treatments [110]. In these cases, it is possible to obtain the long-term benefits of inducing plants to be physiologically resistant to abiotic and biotic stresses while still obtaining the benefit of short-term chemical control.

### 4.3. Effects of Crop Management on the Functioning of Endophytic Symbionts

Understanding holobionts as complex biotic entities enjoins consideration of the crop plants of interest together with their associated microbiomes. We should not think of plants as isolated things having only one genome to be reckoned with. The existence and functioning of plants' microbiomes brings in many other genomes and their expression and effects that influence crop performance.

A recognition of the complexity and dynamics of the natural world blurs any sharp delineation between plants and their microbial associates. Accordingly, holobionts should not be regarded as units functionally separate from the natural and managed environments around them. The myriad relationships between plants and their endophytic symbionts are concurrently influenced by their combined interactions with the biological, physical, and chemical circumstances that impinge upon them.

These wider relationships have not been much studied, but we can report here on research that has investigated the effects of inoculating rice plants with *Trichoderma* before the plants were grown under two different systems of rice crop management [111]. The results showed that the symbiotic interaction between *Trichoderma* and its rice plant hosts was significantly influenced by management practices that modified their growing environment.

The beneficial effects of this fungal endophyte were amplified when combined with the crop management methods recommended for the System of Rice Intensification (SRI), which changes the way that plants, soil, water, and nutrients are managed [112, 113]. The beneficial fungus had more impact with SRI-grown plants, whose inoculation made them EPHs, than on rice plants grown using conventional cultivation practices, that involve the crowding and inundation of rice plants. Trials showed that standard irrigated rice crop management practices diminished or inhibited the beneficial effects of *Trichoderma*.

SRI methods modify the above- and below-ground environments in which rice plants grow. This affects the growth of plants' root systems and also the microbial communities that live around, on, and inside the plants [109, 114–117]. SRI rice paddies are alternately wetted and dried instead of being kept continuously flooded. This reduces crop water consumption by 25% or more at the same time that it boosts production [118,119].

Making the soil mostly aerobic rather than anaerobic (hypoxic) has a major effect on soil microbial populations, of course, but other changes are also involved, such as reducing plant density by 80–90%, from >100/m^2^ to <20/m^2^. These changes are conducive to greater root growth and to more tillering. Modifying the plants' environment affects their morphology and physiology in ways that give substantially more yield even with this much-reduced plant population [111, 119, 120].

SRI promotes organic soil amendments in preference to inorganic fertilization so as to build up the soil's organic matter. SOM is enhanced also by the greater root exudation from larger root systems which increases the substrates for microbial growth. Mechanical weeding that churns up the soil's surface to control weeds also actively aerates the soil. The resulting plant phenotypes are not only more productive but are also more resistant to biotic and abiotic stresses [111]. SRI's cessation of continuous flooding has the additional benefit of reducing methane emissions, with net reductions of greenhouse gas emissions/ha [121, 122].

Two findings of particular interest have emerged from assessing the respective and combined effects of using SRI management practices and inoculating rice seedling roots with a selected *Trichoderma* strain (*T. asperellum* SL2). First, there were very similar effects observed, respectively, from *Trichoderma* inoculation and from SRI crop management methods considering parameters like plant biomass, water use efficiency, and rate of photosynthesis. Second, these effects are significantly greater when both inoculation and SRI management changes are introduced together. The resulting EPH rice plants are more productive, more resilient to stress, and more efficient in their use of resources.

Below are some of the effects on rice plants' performance of modifying management practices (SRI) and *Trichoderma* inoculation (Trich), first separately and then in combination. These effects are consistent with other studies described in this review article as the tables and discussion in previous sections report similar results with other crops and other symbionts (Table 3).

Follow-on research found that rice plants' susceptibility to sheath blight, caused by the pathogenic fungus *Rhizoctonia solani*, was reduced similarly by both *Trichoderma* inoculation and by SRI management methods. But, the reduction in susceptibility was even greater when both interventions were used together. SRI management by itself reduced rice plants' “susceptibility index” to sheath blight infection by 10%; *Trichoderma* inoculation with conventional management reduced the index by 52%. This shows the EPH effect. However, combining the two treatments reduced the index by 68% [124], indicating some synergy between endophytic inoculation and the modified crop management methods. This has been seen in another study, done in Nepal, of the effects of combining *Trichoderma* inoculation with SRI crop management, comparing EPH rice plants with unenhanced plants of the same variety [125].

## 5. Integrated Plant Responses to Root-Endophytic Symbionts Result in Improved Agricultural Performance of EPHs

The endophytic microorganisms described in this paper cause multiple changes in plant gene expression, with frequently improved performance of EPHs compared to plants that lack enhancement with symbiotic organisms. Of course, all plants are colonized by thousands of different microorganisms [12], and some of these can also be beneficial [126, 127], particularly if produced and applied by systems similar to those just described. However, we know that the purposeful application of the four groups of organisms described in this paper, with the appropriate combination of strains and plants under appropriate agronomic practices, provides specifiable benefits at the cellular and molecular levels as discussed below.

### 5.1. Optimized Internal Redox Environment (OIRE) and Resistance to Stress

Plants colonized by the symbionts described here induce specific changes in gene expression [72]. Alterations in plants' gene expression include the enhanced expression of the genes that detoxify reactive oxygen species. Stress on plants, including overexcitation of their photosynthetic pigments, results in the production of ROS which are toxic. The endophytic organisms reviewed here all induce plants to be more resistant to stresses of many kinds, and the mechanisms, proteins, and metabolites that are induced are remarkably similar between these various symbionts (Table 2), even though their lifestyles and modes of plant colonization differ, as discussed in Section 2.

The ROS-degrading enzymes that detoxify ROS include monodehydroascorbate reductase (MDHAR), ascorbate peroxidase (APX), catalase (CAT), and dehydroascorbate reductase (DHAR) [49, 50, 52, 53, 59, 60, 128]. In addition, superoxide dismutase (SOD) acts directly upon the very damaging superoxide radical O_2_^−^, converting it to the less toxic H_2_O_2_ [129]. These several plant enzymes recycle antioxidants, particularly glutathione and ascorbate, from their oxidized form back to their reduced states.

**Figure 3:**
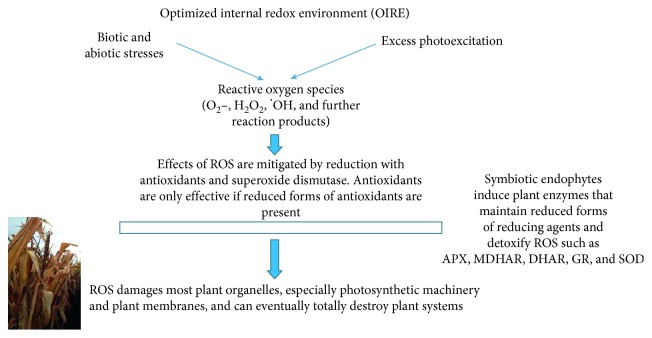
Diagrammatic presentation of how plants and their photosystems are protected from damage by ROS, which is induced by both stress and by photoexcitation. All of the endophytes described in this article have the ability to countervail ROS damage. We hypothesize that this result occurs in better-functioning plants that have optimized internal redox potential.

When antioxidants react with ROS, the latter are oxidized and become inactive until again reduced. All of this lowers ROS levels and neutralizes their adverse effects. In this way, the cells of EPHs maintain an internal redox level that is more conducive to the efficient functioning of their cellular machinery. Such modified cellular systems can be characterized as having an optimized internal redox environment (OIRE) (Figure 3).

The effects of an OIRE help to explain the beneficial effects that microbial symbionts provide to plants [49, 51–53, 58–60]. The antioxidant system in plants operates as a recycling system. If the enzymes for recycling are overexpressed, as they are in the presence of endophytic microbes, especially under stressful conditions, then when ROS levels become high they can be scaled back so that plant cells' internal environment is maintained in a condition where there is balance and complementarity between the chemical processes of oxidation and reduction, summarized in terms of redox potential.

Further, under conditions of drought or salt concentration, plants must protect themselves from losing water into the soil because of osmotic pressure. One method of protection is through the increased production of osmoprotectants, e.g., proline, betaines, and sugars. Several studies have shown that these are increased in the presence of endophytes [50, 128]. All of these mechanisms, including ROS alleviation, are known to be induced in plants by root endophytes.

Throughout this article, we have been discussing EPH increases in the growth of both shoots and roots, plus active plant defenses employed against both biotic and abiotic stresses, and plants' ability to modulate ROS toxicity. All of these functions require the synthesis of new organic compounds and the formation of biological structures. For this, the production of additional proteins, nucleic acids, and other compounds is needed. These several processes are dependent on plants' ability to carry out photosynthesis.

### 5.2. Photosynthesis

To accomplish these various results, photosynthesis must be somehow increased and possibly accelerated. Unless there are increases, none of these results can be achieved since both energy and fixed carbon are necessary for all of the processes described in this review [130].

As suggested above, the activities of endophytic microbes have a crucial role in this. Various methods of measurement have been applied to assess the effect of symbionts on plants' photosynthetic capabilities. These include measuring levels of chlorophyll content and other components of photosynthesis in the leaves, direct measurement of photosynthetic rates, methods gauging chlorophyll fluorescence of dark-adapted leaves (Fv/Fm, performance index), and/or uptake of radio-labeled CO_2_ [131–136]. These results all demonstrate that photosynthesis and the photosynthetic machinery are upregulated in EPHs.

In some cases, photosynthesis is enhanced under nonstressful conditions [22, 123, 128, 133–135, 137] (see also Table 3). But, probably more important is symbionts' ability to reduce plants' loss of photosynthetic capacity under stressful conditions, such as those created by drought and salt [81, 93, 123] or by pathogenesis [51, 54]. These effects are frequently associated with the increased production of enzymes involved in the detoxification of ROS, i.e., they are associated with OIRE [49, 51, 59, 65, 128].

If ROS levels can be kept within a tolerable range, this will result in more efficient functioning of cellular machinery, especially for photosynthesis [138]. Plants colonized with the symbiotic microorganisms frequently are greener [6] and have higher levels of photosynthetic components such as rubisco or chlorophyll, together with higher photosynthetic levels [32, 49, 50, 58, 134].

Both OIRE effects and higher levels of photosynthetic components and greater photosynthetic capability are direct results of the abilities of the symbiotic strains to upregulate genetic expression. In most cases of plants under stress, gene priming appears to take place. In the absence of stress, higher levels of ROS-deactivating enzymes or more photosynthetic components and activity may not be observed. Under stressful conditions, on the other hand, protective/beneficial biochemicals are synthesized. All of these studies and observations are consistent with the proposition that EPHs achieve higher levels of photosynthesis than when microbial symbionts are absent. Greater photosynthesis is required for the colonized plants to exhibit the improved performance that is observed.

### 5.3. Relationship to Plant Shoot and Root Growth

The regulation of ROS levels is not the only factor at work in the increased resistance to stress of plants whose roots have been colonized by endophytic microbes. Above, we discussed the fact that plants with endophyte inhabitants are likely to have larger and deeper root systems. This greater root structure can be expected to allow plants to explore a larger soil volume and to reach down more deeply into the soil system. This enables them to acquire more water from soil under conditions of drought.

Generally, if a plant must provide a portion of its resources to support a symbiotic/commensal organism or to respond to stress, its growth will decrease correspondingly. Similarly, if plants produce larger roots, then their shoot growth is expected to decrease due to competition between roots and shoots for a given amount of photosynthetic and other resources.

The dynamics resulting from microbial agents' induction of resistance to abiotic or biotic stresses all require energy and carbon, and this would seem to be a drain on plant systems. But in fact, usually or at least frequently, greater growth of both shoots and roots is observed in the presence of these endophytes [130]. Endophytic colonization makes plant growth and disease resistance a win-win proposition. The association between EPH plants and their symbionts thus becomes more positive-sum than zero-sum.

There are at least three explanations for this unexpected result: (a) effective endophytes improve the plants' nutrition, either through nitrogen fixation in the case of rhizobia-legume symbiosis or by more effective nutrient acquisition in other cases [6, 25, 31]; (b) many of the proteins and genes that need to be upregulated for beneficial outcomes are activated but then are not expressed until they are needed, through a priming process which makes for more efficient use of available nutrients; and/or (c) photosynthesis is upregulated and protected from damage by the presence and activity of endophytes.

**Figure 4:**
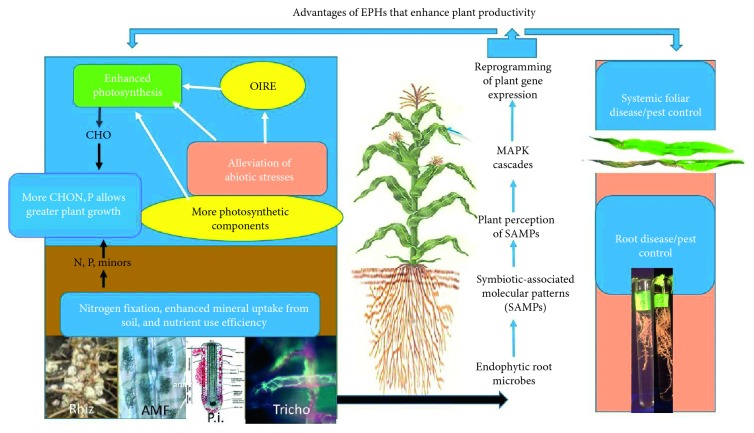
Summary of groups of endophytic microorganisms considered in this paper and summary of mechanisms and systems by which they enhance plant productivity.

In some cases, photosynthesis is enhanced under nonstressful conditions [22, 123, 128, 133–135, 137] (see also Table 3). But, probably more important, we see that symbionts are able to reduce plants' loss of photosynthetic capacity under stressful conditions, such as those created by drought and salt [81, 93, 123] or by pathogenesis [51, 54]. These effects are frequently associated with the increased production of enzymes involved in the detoxification of ROS, i.e., that are associated with OIRE [49, 51, 59, 65, 128]. In most cases of plants under stress, there appears to be some gene priming. All of these studies and observations are consistent with the proposition that EPHs have greater levels of photosynthesis than when microbial symbionts are absent. The greater photosynthesis is required for the colonized plants to exhibit the improved performance that has been noted above. The intricate interactions of symbionts, their SAMPs, and the plants affected are presented diagrammatically in Figure 4.

In this figure, the organisms are shown to colonize the roots of plants (lower center). Their lifestyles within roots differ markedly. Shown are nodules formed by Rhizobiaceae (Rhiz. in the figure) on legumes. They convert atmospheric N_2_ to NH_3_, thereby providing a critical nutrient for plant growth (photo used courtesy of Advanced Biological Marketing).

In the next insert, labeled AMF, are shown arbuscules formed by AMF within infected roots and the hyphae which they form that ramify into the soil, where they are involved in active uptake of P and other nutrients (from [162] and used with the author's permission). The arbuscules provide these acquired nutrients over the arbuscular interface to the plants and, in return, receive nutrients from the plants, including organic compounds.

The third insert shows a diagrammatic representation of root colonization by *P. indica* (designated as P.i.). This fungus colonizes plant roots, initiates programmed cell death, and proliferates in the dead cells just behind the zone of root elongation (designated by red in the figure) (from [163]).

Certain *Trichoderma* strains are rhizospherically and endophytically competent and infect and colonize the cortical regions of roots. Shown here are hyphae of *T. afroharzianum* (designated Tricho) growing endophytically within the root cells of corn [6].

Colonization of roots by these organisms may result in nitrogen fixation (Rhizobiaceae-legumes only), enhanced acquisition of nutrients from soil, and/or increased nitrogen use efficiency. These processes taken together enhance the mineral nutrition of plants.

These organisms also produce SAMPs (Table 1) that interact with plants at the cell membrane level (center section). This results in system-wide signaling to the entire plant and results in changes in plants' gene expression. This, in turn, results in numerous changes in plant physiology. These include resistance to abiotic stresses in part by alleviation of toxicity to ROS through the system that we designate OIRE (Table 2 and Figure 2). This permits more favorable functioning of cellular machinery, including enhancement of photosynthesis. Photosynthesis is also enhanced by the greater expression of photosynthetic components (see section 5.1). Photosynthesis results in production of sugars (designated CHO) that provide the basic carbon scaffolding necessary to form both plant and microbial structures. CHOs are synthesized into more complex molecules that include P, N, and other mineral nutrients from roots. This synthesis results in the formation of amino acids, nucleic acids, lipids including phospholipids (designed CHO, N, P). The energy provided by CHO permits the synthesis of proteins, nucleic acids, and plant structural elements and is essential to enhanced plant growth and development.

Systemic resistance to plant pathogens and pests is also induced, frequently by induction of pathways of resistance. These provide reduction of damage to plants, even in plant parts temporally and spatially separate from the site of application or the location of the endophytic organism or its SAMPs. Shown at far right are leaves of corn grown from a seed treated with *T. afroharzianum* (upper leaf) or without (lower leaf). The leaves were inoculated with the pathogen *Colletotrichum graminicola* (from [164]); similar results have been published with corn treated with *T. virens* or a peptide-based SAMP (from [42, 64]). The disease symptoms are reduced in plants with the symbiont but are not eliminated. Root growth is enhanced, and roots are protected from soilborne pathogens such as *Pythium ultimum* (see insert at lower right).

All of these effects generally result in healthier, more productive plants with increased shoots, roots, and yields.

Higher levels of chlorophyll and the buffering of ROS effects do not tell the whole story, however, because plants grown in the presence of symbiotic endophytes also exhibit some notable morphological changes. They frequently are larger and have more and/or larger leaves. Thus, even if the photosynthetic rates of individual leaves would be similar between endophytic host and nonhost plants, EPH plants' total photosynthetic output will be increased on a per-plant and per-area basis because more leaf area increases the total amount of photosynthesis that occurs over the whole field [56]. This will correlate with more total C being sequestered in the soil, a result that has been confirmed by measurement and that is discussed below in the concluding section on environmental implications.

This relationship has been seen empirically in the first author's studies of corn. The total biomass of corn plants grown in trials was analyzed for C, N, and other elements. On a per-gram basis, the level of C was invariably around 42%. As the yields were increased by *Trichoderma* inoculation and as the crop's root systems became larger, the total C sequestered on a per-area basis became larger.

It is estimated that most plants' total biomass is 2× the amount that can be harvested above-ground [56]. The total level of C was about 13.5 t C/ha in the control plants when both above- and below-ground portions were measured. When the corn seeds planted had been treated prior to planting with a microbial symbiont, the total C was estimated to increase to as much as 25 t C/ha, almost double (one qualification is that the amount of biomass produced was affected by the specific hybrid variety planted, so the combination of symbiont and specific plant variety sown needs to be optimized, as noted earlier. Some hybrids tested gave little or no response to inoculation in terms of the total biomass produced by the plants, which means that they did not increase the total amount of C sequestered from the atmosphere).

In addition to maintaining an OIRE at the cellular level within a plant, plants also must have adequate supplies of nutrients from the soil, including N, P, K, and micronutrients. The rhizobia-legume system provides N through nitrogen fixation; but for other plant crops, this nutrient must be supplied from the soil's reserves or by exogenous fertilizer application. We know that some microbial root endophytes, among their various enhancements, increase the uptake and use efficiency of N [139]. These organisms may also solubilize P and other vital nutrients in the soil and transport them to the roots, thereby enhancing plant nutrition.

AMF are particularly noted for this capability [3], although other microorganisms have similar ability to solubilize P and make this and other nutrients available in the soil that are otherwise poorly available to plants [140, 141]. Photosynthesis is particularly dependent on the plant's sufficiency of N, and the greenness of leaves can be taken as an indicator for the adequacy of N (or lack thereof) in the plant [142].

Recent advances in satellite-based remote-sensing based on measurements of sun-induced chlorophyll fluorescence indicate that some of the most photosynthetically active regions on the planet are in the US Corn Belt. The highly managed cropping system that has been developed there is based on corn plants bred for maximum yield when grown in an intensive manner. The C incorporated into these plants is estimated at >15 g C/m^2^/day (150 kg C/ha/day). If incorporation of C is continued at this level for 60 days, plants would absorb 9 t C/ha/season from the atmosphere [143]. This indicates the feasibility of using an annual crop such as corn to achieve high levels of photosynthesis that can extract C from the atmosphere while also producing a profitable crop yield.

Of course, much or most of the C sequestered into an annual crop may be transitory since it can be re-released as CO_2_ when the crop is harvested, and its biomass is decomposed by animals or microbes. However, the portion of plant biomass that is mobilized into the roots (about half) is not as rapidly degraded, and as discussed elsewhere, it can become stored in the soil as organic matter. Even this portion will eventually decompose, but with an annual crop such as corn, additional biomass is added to the soil each year, thereby increasing SOM over time, and thus bolstering the net amount of carbon in storage.

The corn growth cycle is typically 90–120 days, depending on the variety. Accepting that plant density may vary somewhat, the figure of 9 t of C/ha seems a reasonable average estimate for most corn cropping. This number compares well with the typical corn biomass yields in the US. Good but not remarkably high silage yields in the Corn Belt are 25 tons/acre. Since this is typically 30% dry matter with a carbon content of 42%, this yield represents the sequestration of 7 t of C/ha/season just for the harvested portion of the plant.

Roots also contain about 42% C, so assuming that the plant biomass above and below the ground is roughly equal, the total C fixed in one hectare of land in a season by a corn crop would be approximately 14 t. This is similar to what was obtained with the control plants in the studies discussed above. However, as also seen from the studies cited above, with the most efficient plant-microbe combinations (EPHs), the levels of C that can be fixed can be as much as twice that of plants which are grown without enhanced microbial root colonization.

## 6. Potential Societal Benefits from Greater Use of EPHs

The use of symbiotic endophytic microorganisms to produce EPHs could become a purposeful instrument for improving agricultural production and sustainability and also environmental beneficence and stability. A key to their effectiveness for these purposes is to increase plants' levels and rates of photosynthesis. Thus, endophytic symbionts can enhance photosynthesis and has received almost no attention commercially, although there are many scientific reports on the photosynthetic capabilities in EPHs as reported earlier.

Countries and communities can benefit from more understanding and utilization of the capacities operative in the microbial realm. These are more ubiquitous and have greater effects than previously imagined [144]. The editor of the journal of the American Society for of Microbiology, *MBio*, together with colleagues, has written “Given the extensive influence of microorganisms across our biosphere, we propose that a coordinated, cross-disciplinary effort is required to understand, predict, and harness microbiome function.”

They write further, “From the parallelization of gene function testing to precision manipulation of genes … and development of novel analytical and simulation approaches, strategies need to be developed that move microbiome research into an era of causality. These efforts will improve prediction of ecosystem response and enable the development of new, responsible, microbiome-based solutions to significant challenges of our time” [145].

In this concluding section, we consider how, with further strengthening of our scientific knowledge on the nature and interactions of the microbial agents described here, applications of this knowledge could help to address some of our world's pressing problems, such as hunger and poverty and adverse effects of climate change. Utilizing such knowledge will require a refocusing of research and government initiatives as well as moving farming operations beyond their current overriding focus on food and fiber production, to adopt a dual focus on the production of food and fiber and on protecting and sustaining the world's natural resources and ecosystems. EPHs have the ability, not yet well developed commercially, to

- *Reduce nitrate and nitrous oxide pollution of water* and air through N fixation, by deeper rooting to sequester and intercept N, and by enhancing plants' nitrogen use efficiency (NUE)
- *Mitigate the effects of biotic and abiotic stresses* on plant productivity, which are expected to increase in the future due to climate change and global warming
- *Minimize methane gas production* which contributes to global warming, through use of SRI methods for growing irrigated rice, enhanced by bacterial and fungal symbionts
- *Enhance the sequestration of C from the air* via enhanced photosynthesis and through deeper and greater rooting with more root exudation, transferring C into soil storage, and thereby reducing CO_2_ in the atmosphere as a greenhouse gas that spurs global warming and climate change
- *Contribute to farmers' incomes around the world* by incentivizing their storage of C in the soil though financial mechanisms that provide carbon credits under schemes for C trading or carbon farming concepts
- *Sustain soil productivity into the future* by increasing SOM through the means described in (d), which will make our food supply more secure and our agriculture more efficient and more profitable

The advantages of mitigating biotic and abiotic stresses through symbiotic activity should be exploited, as should the abilities of endophytic microbes to change plants' physiology, such as inducing greater resistance to diseases and pests. Similarly, the ability of endophytes to mitigate the effects of reactive oxygen species, to enhance osmoprotectants, and to activate other mechanisms that can alleviate the effects of drought, salt, and other environmental stresses for agriculture should be capitalized upon. These stresses are likely to increase significantly with climate change, so EPH mitigation of these negative effects is ever more needed. These mechanisms are generally known and can be measured as gene or protein expression in both the lab and in the field, so this is a promising area for academic and commercial development.

### 6.1. Impacts on the Nitrogen and Carbon Cycles

Nitrogen is a vitally important nutrient for all plants. Much of the nitrogen that is currently applied to corn crops as fertilizer is released into the environment, either as NO_*x*_ (N oxide gases) polluting the atmosphere or as nitrates or nitrites that enter the water supply. In the first instance, the N released becomes part of the greenhouse gas accumulation that leads to global warming. In the second, NO_3_- and NO_2_- in ground and surface waters lead to eutrophication of waterways and to the formation of “dead zones” in estuaries where excess nutrient loads from rivers lead to excessive plankton growth and to anoxia where fish and other aerobic organisms cannot survive [146]. Also, concentrations of nitrite in the water supply can have toxic consequences for human beings [147].

To the extent that inorganic N applied as fertilizer is taken up by plants rather than lost by volatilization or leaching, this N is no longer available to pollute the air and the water. If plant roots grow larger and deeper and their nitrogen use efficiency is increased, there will be less pollution of both air and water as more of the available N will be incorporated into plants.

Another effect of having more and deeper roots will be to increase soil organic matter. In SOM, the ratio of C : N is about 10 : 1 [148]. The more N that is incorporated into plants and then decomposed in the soil, the more C (10x more) will also be stored in the soil. And, having more N stored there means there will be less N leaching into the water or being volatilized into the air. Of course, when rhizobia fix N within legumes' nodules, there is direct incorporation of atmospheric N into the plant, which avoids the losses and problems associated promoting plant growth by applying inorganic N fertilizers, of which only 20–30% is taken up by plants.

Worldwide, it is estimated that an increase of 25–50% in root C, together with moderate increases in the depth of rooting, could increase the amount of C stored in the soil as SOM by some 35–100 Mt/yr [148]. This is equal to 80.5 to 230 Mt of atmospheric CO_2_. Withdrawing this much carbon from the carbon cycle would contribute materially to reducing the greenhouse gases that are contributing now to global climate change. The total amount of annual CO_2_ increase in the atmosphere is presently about 16 GT [149]. Such reductions can continue year after year as plant growth continues to be stimulated.

Unfortunately, the measurement of root mass and depth in soil is difficult. In most studies, changes in roots have been studied in greenhouses or on small plants. Reports of root measurements for mature plants are rare. Most measurements of roots require soil removal to a depth of several *m* without destroying the roots, although alternatives are becoming possible. Techniques such as electrode resistivity imaging [150] may be helpful for this. In addition, if soil conditions permit, direct soil coring with quantification of roots extracted [151] may be an option. Further quantification can be obtained by using reporter genes of both microbes and plants and/or by C isotope tagging [152].

### 6.2. Redirecting Agriculture to Countervail the Drivers of Climate Change

Promoting greater rates of photosynthesis that translate into larger and deeper root systems would justify making EPH and other agroecological interventions part of large-scale strategies to mitigate climate change. For this to occur, changes in farming practices and agricultural policy must also take place. We review in this section several kinds of initiatives that could have beneficial impacts on the environment.

According to the United Nations [153], the world needs “an ever-green revolution,” expanded in scope beyond the original Green Revolution that previously improved world food supplies. It is seen from this review that endophytic microbes can change cropping systems in ways that are highly desirable. But, to take advantage of these opportunities, academic, corporate and government systems need to make supportive changes.

Any such system would need continuous verification and validation. The technology noted above to monitor and measure photosynthesis from satellites could provide a major component of an effective verification system [143]. In addition, trials would need to be done to establish valid parameters of increased root growth. Few other efforts to cope with the dynamics of climate change offer so many benefits as the greater utilization of enhanced plant holobionts which capitalize on potentials that already exist in nature and have other benefits beyond climate buffering.

#### 6.2.1. Carbon Farming

Carbon farming is a relatively new concept, referring to the implementation of agricultural practices that are known to improve the rate at which CO_2_ is removed from the atmosphere and converted into plant material and/or soil organic matter, such as agroforestry and conservation agriculture (http://www.carboncycle.org/carbon-farming/).

The strategy is straightforward and simple: CO_2_ from the atmosphere is sequestered in plants, and a substantial share of the resulting fixed carbon is transferred to roots. For carbon farming to function effectively, the rate of photosynthesis needs to be increased, and root biomass, which for example in corn is about 42% carbon, needs to be increased in both density and depth.

Carbon farming can make agriculture a “negative emitter” of greenhouse gases, an oxymoronic designation meaning that this sector removes more C from the atmosphere than it releases. Various practices are now being applied to enhance carbon sequestration and storage in soil, including adding more compost and organic fertilizers to cropped land, increasing undisturbed range planting of perennial grasses, increasing forestation/reforestation and other perennial-plant systems, and enhanced use of cover crops, especially those with deep roots.

According to the Carbon Cycle Institute, “Agriculture is the one sector that has the ability to transform from [being] a net emitter of CO_2_ to a net sequesterer of CO_2_—there is no other human-managed realm with this much potential.” The Institute notes that many current practices for agriculture such as driving tractors, tilling the soil, overgrazing, and using fossil fuel-based fertilizers, pesticides, and herbicides result in significant releases of carbon dioxide emissions. Such practices could and should be modified or curtailed, replaced with other practices that make agriculture more a net absorber of carbon dioxide rather than a producer (http://www.carboncycle.org/carbon-farming/).

#### 6.2.2. Incentivizing C Sequestration

In addition to research and development investments, there would need to be put into place financial systems and provisions whereby the added costs of implementing systems of C sequestration, together with responsible N management, can compensate farmers who make environment-friendly changes in their production systems.

Reliable monitoring as well as changeover to alternative production methods can be fairly expensive. Much of the development of agents and products to produce EPHs has already been done, and they are not particularly expensive [6, 102, 154]. However, the monitoring and validation of effects that is needed to meet cap-and-trade requirements or to qualify for carbon-farming benefits will require some resources.

Further, if the pairing of different varieties of crops with the most effective microbial symbionts is to be optimized, agribio companies will need to be involved. As they are often risk adverse, they will require some incentives for making any far-reaching changes in their production and supply of biological inputs. Introducing changes in agricultural practice on a large scale will require that some appropriate cost-recovery mechanisms be put into place.

Trading of carbon-cap credits and the implementation of carbon-farming practices both need appropriate policy and institutional frameworks. Initially, and for some period of development, revenues would need to be provided to subsidize pilot-scale systems to demonstrate their feasibility. Once they are shown to be successful, their continuation can become part of the global economy because their net economic benefits have been demonstrated to both individual farmers and to policy makers and the public.

Such measurement and reporting could be applied within the systems that are now being established to set caps for carbon emissions and to require net emitters of C to compensate others for any net subtractions of C from the atmosphere that the latter's practices accomplish. There are at least two general approaches to this goal. The first is to compensate farmers through C-cap trading credits for the CO_2_ that they can remove from the atmosphere, and the second is through carbon-farming protocols whereby farmers receive tax credits for demonstrated build up of SOM over time due to their more environmentally friendly farming practices.

In carbon-cap trading, “voluntary greenhouse gas emissions reductions or sequestered carbon by these uncapped entities can be translated into a commodity (i.e., a [certified unit of] carbon offset) which a capped entity (e.g., a coal-fired power plant) can purchase to satisfy its emission compliance requirements if making internal reductions is too difficult and/or cost-prohibitive” [154]. California has implemented carbon-cap trading systems in cooperation with Quebec, Canada. The goal is to put California on a path to meet its goal of returning greenhouse gas emissions to 1990 levels by the year 2020 and to ultimately achieve by 2030 emissions that are only 80% of the 1990 levels [155].

Another approach is to provide tax credits for the implementation of carbon farming as well as other land management practices that reduce and mitigate greenhouse gas emissions on land used in support of farm operations and the further quantification of those benefits using existing state and federal tools for measuring carbon storage in soil and the reduction of CO_2_ emissions [156].

Practices that would achieve these changes include: no-till farming to avoid tillage that results in the oxidation of SOM to CO_2_, adding/returning more compost to the soil, livestock rotation through small paddocks that allow plants to reestablish roots and the soil to incorporate organic matter between grazings, organic mulches that decompose and add organic material to soil, and the use of cover crops that cover the soil to reduce erosion and add organic matter to the soil [157]. Such results can be achieved also by nurturing symbiotic endophytes with intensively produced annual agricultural crops such as corn. The effect is to increase their photosynthesis and also increase their rooting that, year after year, can build up SOM and move atmospheric C into soil storage.

Currently, the market prices offered for C reduction are about $15/t of CO_2_ [155] (http://calcarbondash.org/). We estimate that the promotion of endophytes to establish EPHs could increase the amount of C sequestered by about 12.5 t/ha, with an amount of 46 t of CO_2_/ha removed from the atmosphere (C is only a part of the CO_2_ molecule).

If the net amount of C mobilized into the roots remaining after crop harvest is 40% of this, growers could realize about $275/ha in additional revenue. This should cover the additional costs of verification and still leave farmers with higher net profit as an incentive for adoption. This revenue would be *in addition to* the likely increased value of the grain or silage that they produce from their farming operations because of their higher yields and greater soil fertility.

As an added benefit, both plants and the world would gain from enhanced crop resistance to biotic and abiotic stresses, plus the value of reduced nitrate and nitrous oxide pollution of waterways and the atmosphere, with also healthier plants being grown with the higher levels of soil organic matter. Most of the basic tools to accomplish these goals are available, and pilot-scale studies could be implemented immediately. No genetic engineering or plant breeding that can require many years and large investments of capital would be necessary as with the GMO approaches that have been proposed.

#### 6.2.3. Validation

Efforts to reduce CO_2_ in the atmosphere by sequestration into the soil will require verification and proof. Indeed, the California system specifies certain steps and approaches for validation. These do not presently include the use of symbiotic endophytes or EPHs. For establishing these new activities, the following steps would be required.

- Testing and evaluation should be undertaken to know what are the most effective combinations of plants and microbes to accomplish this goal. If C sequestration as well as biomass and grain production are the goals, then the most effective combinations of corn or other plants plus microbial germplasm need to be identified and validated. As there are tremendous and variable genetic resources in both corn and microbes and with other high-yielding crops as well, this should not be a very difficult task.
- A central objective of any of these systems is to increase carbon storage in the soil as SOM. Therefore, SOM needs to be measured over time as a criterion of efficacy. Soil sampling is a critical factor since deep, medium, and shallow soil horizons all need to be considered.
- Validation of the higher rates of photosynthesis is needed. The development of satellite-based systems to measure photosynthetic rates is already proceeding [143]. This should be linked to the challenge of calibrating enhancement of photosynthesis with correlated growth of roots and greater root exudation as induced by symbiotic root endophytes.
- Validation of levels of total biomass including roots should be done if total C sequestration is taken as a goal. Above-ground biomass is fairly straightforward to measure, but as discussed already, the measurement of roots in mature plants in soil is difficult.

This suite of initiatives will require the types of additional research that have been called for to “enable the development of new, responsible, microbiome-based solutions to significant challenges of our time” [145].

### 6.3. Longer-Term Perspective

What we are describing could be referred to as an adaptation to the theory of “hologene evolution” which was originally proposed by Margulis [158]. In that theory, the formation and inheritance of holobiont systems was considered as a spontaneous natural event. The hologene theory of evolution states “the genome of the host can act in consortium with the genomes of the associated symbiotic microorganisms to create a hologenome. This hologenome … can change more rapidly than the host genome alone, thereby conferring greater adaptive potential to the combined holobiont evolution [159].”

We are proposing that humans have now the information and capability to create hologenomes and interactive microbial systems that can selectively and purposefully create benefits, sustainable and at low-cost, because they are capitalizing upon natural processes and potentials that already exist. These benefits are an extension of the natural order, not some synthetic or exogenous intervention which could have high costs and possibly undesirable consequences.

We humans have an opportunity to introduce endophytic symbionts into cropping systems through seed treatments or other means so as to improve agricultural efficiency and at the same time, achieve positive environmental impacts for the benefit of humankind. Such systems are already being put into place and used on millions of hectares. But, this is only a beginning. There are large opportunities to adapt and extend these symbiotic systems to produce much larger and more favorable impacts on agriculture and the environment than have heretofore been understood and undertaken.

There is an imperative to accomplish these tasks. Rates of climate change are likely to accelerate even if the C emission goals of the Paris accord are met, and further acceleration would result in catastrophic changes for human society. Levels of global warming beyond currently projected levels can result in geo- and biophysical feedback loops that are irreversible, resulting in a “hothouse Earth” scenario with cataclysmic consequences for planet Earth (microbes, at least many of them, would probably survive, but not most other species). Dramatic efforts are needed to reorient our societies, governments, economies, and behavior, to reduce C emissions. Increasing photosynthetic activity sustainably is necessary to reduce the hazards of unbearable warming and to maintain a reasonably stabilized earth.

Under the “hothouse Earth” scenario, sea levels would rise dramatically [160], and significant portions of the Earth would be rendered unhospitable to human life [161]. Avoiding this future requires not only limitations on C emissions, but the removal of greenhouse gases from the atmosphere. The extension of EPHs and the biological systems that we describe here are practical steps to accomplish this goal as well as to make sustainable increases in agricultural productivity despite increasingly inhospitable conditions on Earth.

We know that no single solution will abate and possibly reverse the current adverse trends. Promoting EPH agriculture and carbon farming will not alone prevent the dreadful outcomes that have been foreseen. But, they do hold out the prospect of multiple agronomic, economic, and environmental benefits at reasonable cost. As we come to understand better the potency of many kinds of microbiomes, we should not forgo these opportunities because we do not recognize and utilize our interdependency with the microbial realm.

## Figures and Tables

**Table 1:** Examples of the abilities of endophytic symbiotic microorganisms and their SAMPs to increase plants' productivity and yield.

Symbiotic microbes	Crop	Effects
Rhizobiaceae	Soybean	Meta-analysis showed −6 to 176% increase in soybean yields across 28 studies [43].

Commercial *Rhizobium* inoculants	Soybean	On farmer fields in Michigan, yields were increased by 23–45% where inoculants had not been used previously. Average yield increased 2-3% where inoculants had previously been used [44]. In Indiana, yield increases were ∼1.5–2% [45].

*Rhizobium*	Common bean	Increases of 2–3.5 t/ha under dry conditions [16].
*R. leguminosarum* bv. *trifolii*	Rice, wheat, and corn [17, 19, 46]	Increases in yield were seen under field conditions. With corn, not all plant genotype-microbial combinations increased yield.

AMF	Numerous crops	Across numerous studies in the literature, AMF inoculation has resulted in increases in yield but not statistically different from zero. In grasses, the combination of aerially applied endophytic fungi and AMF gave greater than expected results than from either alone [47, 48].

AMF (*Glomus versiforme*)	Watermelon	Increased shoot and root growth seen compared to untreated controls in drought but not well-watered conditions. Inactivation of reactive oxygen species (ROS) by gene expression changes was required [49, 50].

*Piriformaspora indica*	Over 150 plant species	Various studies have identified plant growth-promoting activities of plants whose roots were colonized by *P. indica*, as reviewed [8]. Improvements in plant performance include better seed germination under temperature [8], improved resistance of plantlets during micropropagation [31], and stress resistance.

*P. indica*	Barley	*P. indica* reduced effects of stresses and pathogens, inducing reprogramming of plant gene expression, which resulted in increased plant biomass and resistance to abiotic stresses [51]. These include upregulation of enzymes that inactivate toxic levels of reactive oxygen species (ROS) that are formed in plants under stress [50, 52–54].

*Trichoderma afroharzianum, T. virens, T. viride*, and other species	Numerous plant species	Inoculation with the organism induced increased growth responses in numerous vegetable species [55], greenhouse ornamental plants [6, 7], and cereal crops [6, 7, 56, 57].

*T. afroharzianum*	Tomato, corn	Seed treatments applied to corn or tomato resulted in endophytic colonization of plant roots. This colonization is associated with increased resistance to stresses and is causally associated with higher levels of expression of enzymes that inactivate ROS [58–60].

*T. afroharzianum*	Grapes	Application, even to the soil, increased fruit yield and increased total amount of polyphenols [61].

SAMPS	Derived from:	

Chitooligosaccharides (COs) and lipochitooligosaccharides (LCOs)	Rhizobiaceae and AMF [62, 63]	Increased seedling growth of roots; increased yields of corn and other crops including leaf area, shoot mass, and root mass; root branching; increased photosynthesis; changes in plant gene expression; induced resistance to plant diseases. LCOs are produced by the bacteria, but COs may elicit similar plant responses. These compounds added to plants of many kinds result in season-long disease resistance and plant yield increases [34, 36].

6-Pentyl-*α*-pyrone (6PP)	*T. afroharzianum*	Application of this volatile unsaturated lactone molecule, even to the soil, increased fruit yield and increased the total amount of polyphenols as effectively as did treatments with the organism [61].

1-Octen-3-ol (1o3)	Various *Trichoderma* spp.	Seed treatments with picoliter quantities of this volatile metabolite resulted in season-long improvements to shoot and root growth in corn as effectively as did treatments with the fungus itself [56].

Harzianic acid (HzA)	Various *Trichoderma* spp.	This has both antifungal and growth promotive activities and acts as a siderophore to chelate iron [39].

Peptabiols (Pb)	Various *Trichoderma* spp.	These induced plant defense responses and are inhibitory to soil microflora. These are peptides, and hundreds of separate compounds have been identified [40].

Hydrophobins and other hydrophobin-like proteins (Hp)	Various *Trichoderma* spp.	These hydrophobic proteins induce plant resistance and increase plant growth [41, 64]. There is great variability between these proteins, and only a few have beneficial activity.

Plant response-like protein	*T. formosa*	Induces immunity to a virus, a fungus, a bacterium, and an oomycete plant pathogen [65].

**Table 2:** Examples of control or inhibition of plant pathogens or pests by endophytic plant microbes.

Disease or pathogen	Symbiont	Plant	Response of plants to endophytes	Reference
Numerous soil pathogens, including *Fusarium*, *Rhizoctonia*, *Sclerotinia*, *Macrophomina*, and *Cylindrocladium*	Various rhizobia	Legumes, including soybean, chickpea, pea, lentil, lupine, and fava bean	Control of many pathogens	[84]
*Phytophthora cinnamon*	Bradyrhizobium japonicum	Soybean	The bacteria also colonized the pathogen	[85]
*Sclerotinia sclerotiorum* (white rot)	*Mesorhizobium loti*	Indian mustard	Nearly complete control of white rot	[86]
Powdery mildew	AMF (*Funneliformis mosseae*)	Barley	Induced resistance gave a high degree of control	[87]
Tomato leaf curl virus	AMF (*Funneliformis mosseae*)	Tomato	Systemic resistance reduced disease severity	[88]
Phytophthora late blight	AMF (*Rhizophagus irregularis*)	Potato	Symptoms reduced, but not under conditions of high disease pressure	[78]
*Spiroplasma citri*	AMF (*Glomus mosseae*)	Madagascar periwinkle	Control occurred, but another pathogen was not controlled	[79]
Various nematodes	AMF	Various plants in summary	Wide variety of plants are protected against these round worms	[80]
Numerous root rot pathogens including *Verticillium*, *Fusarium* foot rot, *Rhizoctonia*, and *Thielavopsis*	*P. indica*	Various plants and pathogens in summary	Wide variety of plants protected against these fungal pathogens	[31]
Fungal diseases of leaves, including *Alternaria brassicae*, *Botrytis cinerea*, and powdery mildew	*P. indica*	Chickpea, barley, and others	Disease control of above-ground parts even though symbiont only in roots; antioxidant systems are important	[51, 89]
*Fusarium* head blight	*P. indica*	Wheat	Grain disease occurred even though symbiont was only in roots; also reduced *Fusarium* mycotoxin production	[90]
Various root and foliar pathogens	*Trichoderma* spp	Various plants	Numerous examples of control of pathogens in roots and above-ground plant organs	[7]
Various root nematodes	*Trichoderma* spp	Tomato	Control occurred with systemic control demonstrated using split-root plants	[56]

**Table 3:** 

	Seedling root biomass (g)	Seedling canopy biomass (g)	Rate of photosynthesis (*µ*mol·m^−2^·s^−1^)	Stomatal conductance (mmol·m^−2^·s^−1^)	Internal CO_2_ concentration (ppm)	Chlorophyll *a* (mg/g)^*∗*^	Panicle number	Filled grains (%)
Trich w/std mgmt	14.25b	12.68c	5.19b	527.99b	376.93a	1.34b	8.93b	58.33b
SRI w/o Trich	16.72ab	15.46b	5.15b	513.91b	364.10a	1.44b	8.66b	61.20b
SRI + Trich	23.75a	21.38a	7.81a	827.31a	314.39b	1.96a	12.73a	88.00a

^*∗*^There were no significant differences for chlorophyll *b* in the leaves Source: [123]. Treatments and methods are explained in that publication.
